# Some Dangers of Milk1Read before the Vigo County Medical Society.

**Published:** 1898-01

**Authors:** T. B. Pote

**Affiliations:** Sanitary Inspector to the Board of Health of Terre Haute, Indiana


### SOME DANGERS OF MILK.[1]

By T. B. Pote, D.V.S.,
SANITARY INSPECTOR TO THE BOARD OF HEALTH OF TERRE HAUTE, INDIANA.

Milk is an opaque, white fluid secreted from the mammary gland of the female sex of the mammalian class, commencing immediately after the birth of its young, and continuing throughout the period in which the young is too immature to subsist upon the food of the adult.

Throughout the history of man, from his most primitive state, it is shown that this secretion of animals has been made use of as an article of food, and in different parts of the world the milk of several different domestic animals has been used, but at the present time the milk of the cow is that in general use throughout the civilized world.

The enormous amount of this secretion consumed as food may be realized when we estimate that there are in the United States alone about 17,000,000 milk-cows furnishing milk and milk-products, but 5,000,000 cows alone supply more than a billion gallons of milk to consumers annually.

This secretion of milk by animals was one to attract the attention of the early philosophers, and many curious theories and doctrines held by them have been handed down to us, but at the present they are of value only in so far as they go to make up curious history of the subject.

[1] Read before the Vigo County Medical Society.

Advancements in physiology and chemistry have been the means of bringing to light its true origin and composition and its true value as an article of food. It is a food containing all the elements required for the growth of the body, and is a perfect food for the young, being the only article supplied by nature which combines all the elements requisite to secure healthy nutrition in a form suited to the young. Containing as it does the three classes of principles, in association with water, viz., the albuminous or nitrogenous, the oleaginous, and the saccharine, it is a splendid food for the adult human being as well as for the young.

The study of milk bacteriologically has shown it to be a fluid which is almost as perfectly adapted to the existence of bacterial forms of life as it is as a food to the higher organisms.

Milk in the udder of the cow is a sterile fluid, providing the cow is a healthy one, but the moment it reaches the atmosphere it becomes the soil of bacteria, which increase rapidly on standing exposed to the air at a normal temperature.

Commercial milk, exposed to ordinary conditions, shows from 10,000 to 100,000 bacteria to the c.c.; but, fortunately, most of these are harmless, and, in fact, milk contains some bacteria which are said to add to its flavor, etc. On the other hand, however, there are those in which the elements of danger are evident, only waiting their opportunity to commence their fearful work of destruction.

Danger lies in the consumption of the fluid in which there are numerous bacteria, since if they are present the conditions are favorable for any and all forms, and among the many present and harmless there may be the few which are dangerous and destructive.

Bacteria being omnipresent, and milk being peculiarly suited to their growth, it necessarily follows that it gains these low organisms through the medium in which it passes to the consumer. In the dairy we have, therefore, come to know that the conditions under which milk is produced and handled in its preparation for the market affect it very materially. In commercial milk may be found the cause of many ailments which the human being is heir to, some sporadic in their nature, while others are contagious. The improper cleansing of vessels in which milk is handled, or the medium in which it may be allowed to remain, may result in the admission of bacteria which may produce an effect upon the milk, ending in the production of some ill effect in the digestive organs of the delicate child or invalid.

The splendid soil which milk forms for bacteria, and the slow cooling process frequently found in use among dairymen, allow the bacteria to produce sometimes, at a rapid rate, spores or ptomaïns which may often be found far more dangerous than the bacteria themselves.

Germs of disease, coming from the diseased condition of the cattle supplying milk, mark one of the greatest dangers to the consumer of milk, because of the fluid usually entering the stomach as a raw food. Of such germs may be noted the bacilli of tuberculosis, a disease most prevalent in cattle, and especially dairy-cattle, affecting all animals and destroying more human lives annually than diphtheria, scarlet fever, typhoid fever, and smallpox combined. More than 14 per cent. of all deaths that occur (one person out of every eight, or, under some conditions, one in every three), are the result of this disease. It is prevalent throughout the world, and in the United States alone it is estimated that of the 63,000,000 people, 9,000,000 or more will die of tuberculosis unless something be done to prevent it. It is the opinion of an authority that the bacilli of tuberculosis is responsible directly, or indirectly, for not less than 150,000 deaths annually in the United States. In our own city 18.33 per cent. of all deaths occurring in 1896 were due to this disease, a greater percentage of mortality than the combined deaths form diphtheria, scarlet fever, typhoid fever, and smallpox, the contagious diseases so dreaded.

The exact percentage of cattle affected with this disease is difficult to reach, owing to the difficulty of collecting data upon the subject. However, wherever systematic inspection of cattle is being carried on, the percentage of dairy-cattle found tuberculous is alarmingly high. In this country an estimate is made that 5 per cent. are affected. Individual herds are not few where they have shown from 40 to 90 per cent. of their number affected with this disease. A herd of cattle supplying milk to this city, a few days ago, showed 76⅔ per cent. of its members suffering from this disease in the most advanced state, other herds having shown a lesser percentage, from 5 to 10 per cent..

Thus is presented one of the dangers of milk to the adult, and especially the infant—milk bearing the bacilli entering the system in a raw state, as before mentioned. Milk enters largely into the dietary of children. A large percentage of infants to-day are bottle-fed, and this proportion is increasing; therefore, it seems reasonable to conclude that the great mortality of children under five years of age may be largely due to infected milk, and especially to tubercular infection by way of the intestinal glands by ingestion of milk.

Besides tubercular infection from milk, there are other dangers of disease through the agency of milk.

Typhoid fever has many times been scattered broadcast through the medium of milk. Such a danger lies in the use of milk, should it come from an infected district : 1st, in that the bacilli may gain admittance to the milk from a person working with it suffering from a mild form, or the so-called ambulatory form, or perhaps one having the care of milk and nursing a typhoid patient; 2d, a great danger lies in the possible adulteration of milk, which might be contaminated with the bacillus causing typhoid fever. The typhoid bacillus, once in the milk, finds it a splendid soil for its development, and germinates at a rapid rate, one little bacillus being sufficient to impregnate a whole can of milk, spreading the disease to a whole village or neighborhood.

The transmission of scarlet fever and diphtheria may also take place through the medium of milk, and, while these transmissions are not so well understood, they are nevertheless quite evident.

By some it is held that cows suffer these diseases and that the milk from them conveys the disease to man, but positive proof of such is wanting.

The health of the dairyman and his family, as well as any who may have the care of milk, constitutes an important factor relative to the dangers of milk from the diseases which have been mentioned. Bacteria, in their process of growth, produce spores or sometimes ptomaïns which may not affect the adult using the milk, but surely affect the health of the young child whose delicate organism has not developed to the point of becoming accustomed to the poison, and, therefore, cannot react against it as would be the case in the adult.

A more severe and dangerous ptomaïn-poison is that of tyrotoxicon, a poison thought to be due to some micro-organism, or resulting from fermentation of some form. The poison may be exhibited in milk, cheese, or ice-cream, often coming to light in a wholesale ice-cream poisoning. It is chemically a diazbenzol, crystallizing in six-sided plates, producing symptoms of dryness and constriction of the fauces, nausea, nervous depression, etc.

There are other dangers to which attention might be directed, but are often overlooked by reason of no immediate effect being shown. Such is adulteration, and particularly in the line of preservative preparations which are used for their preservative effect.

These preparations contain, as a basis, borax, boric acid, salicylic acid, or formalin, and we can readily understand the effect of these drugs upon the system of the delicate child or invalid, especially when taken day after day.

There are many vital points to this subject of milk-supply, and it is one commanding much attention at the present time. Bacteriology has thrown a new light upon the question of dairying and public milk-supply, and new dangers confront us from this point as we grow older as a people. In our advancement in civilization we leave off getting our milk from a few individual cows which roam the broad fields of pasture-lands to seek the food and exercise which best suit their tastes and needs. They now are confined in close barns, often not allowed the necessary amount of exercise, are in unhygienic conditions as to surroundings, and fed on poor food far from that which nature intended for them. Added to this, the forcing system carried on by many dairymen must necessarily make the cow another animal. Along with this the changes in the care of milk, by reason of supplying large numbers of consumers from one depot, instead of as formerly, supplying only a few from one place, account in great part for one of the present dangers of milk.

